# Supplementary material for: Small RNAs as biomarkers to differentiate benign and malign prostate diseases: An alternative for transrectal punch biopsy of the prostate?
Source: PLoS One. 2021 Mar 24;16(3):e0247930. doi: 10.1371/journal.pone.0247930 (PMC7990312; doi:10.1371/journal.pone.0247930)
Supplement: Attachment — Submitted filename: Response to Reviewers.SmaRNAdiMP.docx [file pone.0247930.s001.docx]

Response to Reviewers

Dear Sir or Madam!

Thank you very much for your most kind reply with all the helpful questions and comments on our paper. I thank you for the opportunity to respond and would be pleased if the answers are satisfactory to you.

In the following, I will address the individual points, highlighted in color. In addition, you will receive both the marked and the unmarked version of the reviewed paper.

In response to your mail received on January 21^st^ 2021:

1. Please ensure that your manuscript meets PLOS ONE's style requirements, including those for file naming. The PLOS ONE style templates can be found at

https://journals.plos.org/plosone/s/file?id=wjVg/PLOSOne_formatting_sample_main_body.pdf and

https://journals.plos.org/plosone/s/file?id=ba62/PLOSOne_formatting_sample_title_authors_affiliations.pdf

The layout was changed to fit the specifications. Pardon for the previous inconsistencies.

2. In your Methods section, please provide additional information about the participant recruitment method and the demographic details of your participants. Please ensure you have provided sufficient details to replicate the analyses such as: a) the recruitment date range (month and year), b) a description of how participants were recruited.

The information was specified in the text. (line 147-174)

3. Please provide a sample size and power calculation in the Methods, or discuss the reasons for not performing one before study initiation.

Due to the absence of preliminary studies on this topic, only a qualified estimate could be made. This has now been explained in the text.

4. Please ensure you have discussed any potential limitations of your study in the Discussion, including study design, sample size and/or potential confounders.

The limitations of the study were named in more detail. (line 479-486)

5.We note that you state that consent was obtained from legal guardians in some cases. Please explain why patient consent was not appropriate in some cases. Please describe in your methods section how capacity to consent was determined for the participants in this study. Please also state whether your ethics committee approved this consent procedure. If you did not assess capacity please briefly outline why this was not necessary in this case.

According to German guardianship law, legally appointed guardians make decisions for their personally known clients in certain medical cases. The prerequisite for the appointment of a guardian is that the person concerned is of adult age and in need of assistance. A person is in need of assistance if, as a result of illness or disability, he or she is no longer able to manage his or her affairs in whole or in part. Of course, in this case, client and legal guardian were asked for written consent.

6. Please provide additional details regarding participant consent. In the ethics statement in the Methods and online submission information, please ensure that you have specified what type you obtained (for instance, written or verbal, and if verbal, how it was documented and witnessed).

Written consent was always obtained. This has now been specified in the text. (line 144)

7. We note that you have indicated that data from this study are available upon request. PLOS only allows data to be available upon request if there are legal or ethical restrictions on sharing data publicly. For information on unacceptable data access restrictions, please see <http://journals.plos.org/plosone/s/data-availability#loc-unacceptable-data-access-restrictions>.

The relevant raw data will be gladly made available in its entirety. It is submitted at biostudies (<https://www.ebi.ac.uk/biostudies>) with the number S-BSST586.

In your revised cover letter, please address the following prompts:

a) If there are ethical or legal restrictions on sharing a de-identified data set, please explain them in detail (e.g., data contain potentially identifying or sensitive patient information) and who has imposed them (e.g., an ethics committee). Please also provide contact information for a data access committee, ethics committee, or other institutional body to which data requests may be sent.

There are no objections to the disclosure of the study data. Clear names are not mentioned in it. Regarding all data questions, the Ethics Committee of the University of Witten/Herdecke can be contacted via sekretariat-ethik@uni-wh.de.

b) If there are no restrictions, please upload the minimal anonymized data set necessary to replicate your study findings as either Supporting Information files or to a stable, public repository and provide us with the relevant URLs, DOIs, or accession numbers. Please see http://www.bmj.com/content/340/bmj.c181.long for guidelines on how to de-identify and prepare clinical data for publication. For a list of acceptable repositories, please see <http://journals.plos.org/plosone/s/data-availability#loc-recommended-repositories>.

We uploaded the complete relevant data at: biostudies (<https://www.ebi.ac.uk/biostudies>) with the number S-BSST586

We will update your Data Availability statement on your behalf to reflect the information you provide.

8. Please amend either the title on the online submission form (via Edit Submission) or the title in the manuscript so that they are identical.

The title was adapted in the manuscript.

9. Your ethics statement should only appear in the Methods section of your manuscript. If your ethics statement is written in any section besides the Methods, please delete it from any other section.

The text has been adjusted accordingly.

10. We note you have included a table to which you do not refer in the text of your manuscript. Please ensure that you refer to Table 4 in your text; if accepted, production will need this reference to link the reader to the Table.

The numeration mistake has been corrected. (line 139-141)

11. Please include a copy of Table 5 which you refer to in your text on page 12.

 Meant was Table 4, as has now been correctly adjusted.

Moreover, an honest attempt was made to address all of the reviewer´s comments. They were very helpful and seemed fair to us.

Reviewer's Responses to Questions
> 
> Comments to the Author
> 
> 1. Is the manuscript technically sound, and do the data support the conclusions?
> 
> The manuscript must describe a technically sound piece of scientific research with data that supports the conclusions. Experiments must have been conducted rigorously, with appropriate controls, replication, and sample sizes. The conclusions must be drawn appropriately based on the data presented.
> 
> Reviewer #1: Partly
>

We have made another effort to disclose the background behind the study and the statistics. We have also tried to make the summary more specific to the results.
> ________________________________
> 
> 2. Has the statistical analysis been performed appropriately and rigorously?
> 
> Reviewer #1: Yes
> 
> ________________________________
> 
> 3. Have the authors made all data underlying the findings in their manuscript fully available?
> 
> The PLOS Data policy<<http://www.plosone.org/static/policies.action#sharing>> requires authors to make all data underlying the findings described in their manuscript fully available without restriction, with rare exception (please refer to the Data Availability Statement in the manuscript PDF file). The data should be provided as part of the manuscript or its supporting information, or deposited to a public repository. For example, in addition to summary statistics, the data points behind means, medians and variance measures should be available. If there are restrictions on publicly sharing data—e.g. participant privacy or use of data from a third party—those must be specified.
> 
> Reviewer #1: Yes
>

We have now made all the necessary raw data public. As a consequence, we excluded supplementary data (supporting information) with the manuscript in order to avoid duplication.
> ________________________________
> 
> 4. Is the manuscript presented in an intelligible fashion and written in standard English?
> 
> PLOS ONE does not copyedit accepted manuscripts, so the language in submitted articles must be clear, correct, and unambiguous. Any typographical or grammatical errors should be corrected at revision, so please note any specific errors here.
> 
> Reviewer #1: Yes
> 
> ________________________________
> 
> 5. Review Comments to the Author
> 
> Please use the space provided to explain your answers to the questions above. You may also include additional comments for the author, including concerns about dual publication, research ethics, or publication ethics. (Please upload your review as an attachment if it exceeds 20,000 characters)
> 
> Reviewer #1: In this paper, authors investigate wether urinary miRNAs and piRNAs can be used as biomarkers to differentiate benign and malign prostate diseases. This should be an alternative to transrectal punch biopsy of the prostate which is a painful and risky procedure.
> 
> For that, urine samples from urological patients with BPH (25) or PCa (28) were analysed using Next-Generation Sequencing to detect the expression profile of total and exosomal miRNA/piRNA. Then a differential study was made and 6 miRNAs and 2 piRNAS were statistically validated
> In addition, machine-learning algorithms were used to identify a panel of 22 additional miRNAs.
> 
> This paper is of interest and well written in its entirety.
> The differential study is clear and robust and conclude to a set of potential biomarkers.
> However, the machine-learning part is less clear and its interest is not an evidence for me.
> 
> MAJOR remarks and questions:
> 
> 1) In the paper, samples were split randomly into training dataset (18 PCA + 15 BPH) and a test dataset (10 PCA + 10 BPH) for validation. Sizes of dataset were not justified and it is difficult to know the relevance of this choice. It would be necessary to justify this choice.
>  (line 181-197)

Since there have been no previous studies on this concrete issue, we were missing the variances of the individual miRNAs and the expected differences between the two groups. So, we could not make a valid power-calculation in advance and relied on the assessment of the resident statisticians (n>40). We are well aware of the fact that the small sample size is a legitimate concern. However, this study was clearly explorative in character. Its aim was to provide a first experimental ground on which further investigations can be built up.

> 2) The interest of additionnal biomaker panels found with machine learning method is not evidence for me. This should be clarified and the interest of such a method be clearly shown.
> (line 463-478)

If the candidates of the pattern turn out to be more amplification-stable or particularly more pathway-relevant than the individual markers, a panel and disease-specific expression pattern could be highly relevant for further research. Furthermore, such a pattern is probably less susceptible to influences on the expression and analysis of individual markers. Consequently, a diagnostic test based on it may be more reliable.

Nevertheless, the single markers can also be seen as promising approaches. both avenues should be further investigated.
> 
> MINOR remarks:
> 
> lines 115-116: maybe only use miRNA and not microRNA - was revised
> line 166: the reading depth -> maybe reading length - was revised
> line 166: sentence is not really clear - was revised
> Figure 2: not centered and truncated - was revised
> Figure 6: problem of legend for PCA and PH - was revised
> 
> ________________________________

Also, all figures were checked and adapted with pace.com.

I am pleased about the cooperation and would still be very happy about the publication at PLOS.

If any further questions arise, I am available at any time.

Kind regards from Witten!

Lukas Markert
